# Standardised mortality rate for cerebrovascular diseases in the Slovak Republic from 1996 to 2013 in the context of income inequalities and its international comparison

**DOI:** 10.1186/s13561-016-0140-4

**Published:** 2017-02-02

**Authors:** Beáta Gavurová, Viliam Kováč, Tatiana Vagašová

**Affiliations:** Němcovej 32, Košice, Slovakia

**Keywords:** Standardised mortality rate, Cerebrovascular diseases, Income inequality, Regional disparities, the Slovak Republic

## Abstract

Non-communicable diseases represent one of the greatest challenges for health policymakers. The main objective of this study is to analyse the development of standardised mortality rates for cerebrovascular disease, which is one of the most common causes of deaths, in relation to income inequality in individual regions of the Slovak Republic. Direct standardisation was applied using data from the Slovak mortality database, covering the time period from 1996 to 2013. The standardised mortality rate declined by 4.23*%* in the Slovak Republic. However, since 1996, the rate has been higher by almost 33% in men than in women. Standardised mortality rates were lower in the northern part of the Slovak Republic than in the southern part. The regression models demonstrated an impact of the observed income-related dimensions on these rates. The income quintile ratio and Gini coefficient appeared to be the most influencing variables. The results of the analysis highlight valuable baseline information for creating new support programmes aimed at eliminating health inequalities in relation to health and social policy.

## Background

The health status of a country’s population is the result of a complex interplay of genetic features, socio-economic situations, and environmental, nutritional, and lifestyle factors, as well as of the general availability of health care including preventive programmes [1]. The data on mortality are common indicators used to measure and compare a country’s health status at the local, national, and international level, as these data are regularly and widely collected. Understanding the health status of a population should form the baseline for establishing effective health policies, allocation of funds, and prioritisation of health care in the country [2–4]. The Slovak Republic has currently developed proposals for reforms in the health sector, and these changes should be based on relevant analyses concerning the health status of a population and demographic indicators. In terms of mortality, it is desirable to examine the leading causes of death because these conditions require increased attention from health policymakers in the Slovak Republic.

The most common causes of death include cardiovascular diseases, which cause approximately 45% of all deaths in the Slovak Republic. In particular, ischaemic heart diseases and cerebrovascular diseases are the leading causes of mortality in the Slovak Republic [5].

The World Health Organization defines cerebrovascular diseases as rapidly developing clinical signs of focal cerebral dysfunction that last for more than 24 hours or lead to death without the presence of an apparent cause other than cerebrovascular malformations. Ischaemic stroke is defined as blockage of the blood vessels affecting tissue in the central nervous system. Unlike transient ischaemic attack, ischaemic infarction may be symptomatic or asymptomatic. Cerebrovascular diseases are a predominant cause of death in developed countries [6]. They present long-term and significant socio-economic issues worldwide [7–9]. The incidence of transient ischaemic attacks in Europe and in the United States is estimated to range from 0.37 to 1.1 per 1,000 inhabitants per year, which exponentially increases with age regardless of race and gender from 6 to 16 per 1,000 inhabitants aged 85 years and older per year [10]. Its prevalence is estimated to range from 0.4*%* to 4.1*%*. In 2002, experts had already examined the development of the mortality rate due to cerebrovascular diseases in Europe, the United States, and in Japan [11, 12]. Their studies confirmed that the worst results at that time occurred in eastern European countries. With the increasing life expectancy as well as the number of elderly, a rise in the prevalence of cerebrovascular diseases can be expected in many developed countries.

Despite the advanced multidisciplinary research on cerebrovascular disease in many countries, respective analyses are absent for the Slovak Republic. The available partial analyses of medical fields assess the country’s population health issues separately, in the context of outputs from various casuistries. They do not provide comprehensive or relevant baselines for health and social policy, because they lack a contextual link with demographic processes such as the ageing of the population and correlations with socioeconomic characteristics and regional disparities in health. Knowledge of these determinants, the causal relations, and their quantification should be incorporated into targeted research studies and would provide important information for many policymakers as well as for the creation of targeted metrics representing the health status of the Slovak population within the strategic framework of the healthcare system. A systemic problem that occurs with prevention programmes is that if they are not properly targeted or are implemented with inappropriate tools, they lose their primary effect, and thus efforts to increase the effectiveness of the health system do not achieve the desired countrywide results.

The situation outside of the Slovak Republic is far more positive. Multidimensional analyses of mortality and its determinants have long been at the heart of many disciplines, including the medical, economic, social sciences. This is clearly evidenced by the quality of the available epidemiological scientific research studies [13, 14] as well as the further development of similar fields [15–20]. In addition to scientific research institutions, the issue of morbidity and mortality is also explored by international institutions – for instance, the World Health Organization, Eurostat, and the Organisation for Economic Co-operation and Development [6].

The aim of this study is to identify the most vulnerable groups of people in the past 17 years and to find the regions of the Slovak Republic with the highest cerebrovascular disease mortality rate while considering the income inequality between regions.

The main objectives of this study are as follows:

- To analyse the progression of mortality rate due to cerebrovascular diseases in the Slovak Republic in comparison with selected European countries;
- To reveal regional differences, both in the level and progression of the cerebrovascular disease mortality rate, in the Slovak Republic;
- To quantify the relationship between the mortality rate and income indicators in the individual regions of the Slovak Republic.

The main contribution of this study is the identification of regional discrepancies in the mortality rate of cerebrovascular diseases in the Slovak Republic and the analysis of their reasons in relation to each region’s income indicators. When examining the relationship between mortality and income indicators, we expected to observe a positive linear relationship between mortality and the explored indicators – unemployment rate, poverty, Gini coefficient, social benefits, and income quintile ratio. On the contrary, a negative linear relationship was expected for disposable income.

## Methods

The data and methodology section offers an overview of the applied dataset and methodology.

### Data

The mortality rate was computed from data on the number of deaths due to cerebrovascular diseases – marked as codes *I*60 to *I*69 – according to sex, five-year age groups and individual regions of the Slovak Republic from 1996 to 2013. Under the conditions of their contract, the National Health Information Centre (Národné centrum zdravotníckych informàcií) of the Slovak Republic provides a primary source of data on national health statistics.

Mid-year population data by sex, age group and individual region for all explored years were obtained from the Statistical Office of the Slovak Republic (Štatistický úrad Slovenskej Republiky). Mortality rate was age-standardised to the revised European standard population by the age groups adopted by Eurostat in the last revision in 2012. For international comparisons, countries with extreme cerebrovascular disease mortality rates were selected, as were countries of the Visegrad Group – the Czech Republic, Hungary, Poland, and the Slovak Republic, which show similarities due to their shared post-socialist development. The data were available from 2004 to 2012.

In particular regions of the Slovak Republic, we tested the relationship between the standardised mortality rate and the following socio-economic indicators:

- Unemployment rate – expressed as the share of unemployed inhabitants of the number of economically active inhabitants in the previous year;
- Disposable income of a household – the mean equivalised net income per household – expressed in EUR per month;
- Poverty – the share of the population with an income lower than the at-risk-of-poverty threshold compared to the whole population;
- Gini coefficient;
- Income quintile ratio – *S*80/*S*20 ratio;
- Social benefits – the amount of all social benefits received by an individual – expressed in EUR per month.

The data on unemployment rate were obtained from the Statistical Office of the Slovak Republic, and the other indicators were downloaded from the European Union Statistics on Income and Living Conditions, which is the most extensive statistical survey on income, living conditions and poverty indicators in the European Union from 2004 to 2013.

There are a few notes to consider regarding the definition of the chosen income indicators. The unemployment rate indicates the ratio of the number of unemployed inhabitants out of the number of economically active inhabitants in the previous year. The mean equivalised net income per household represents the household disposable income divided by the equivalent household size. Individual household members are assigned weights – 1 for the first adult household member, 0.5 per each additional adult member, 0.5 per each adolescent 14 years of age and over and 0.3 per each child younger than 14 years of age. The at-risk-of-poverty threshold is set at 60% of the national median of individual equivalised disposable income. It expresses the percentage of inhabitants with an equivalent disposable income below a set boundary.

The Gini coefficient is an indicator of monetary poverty that represents inequality in income distribution and is defined as the relationship of cumulative shares of the population arranged according to the level of equivalised disposable income compared to the cumulative share of the equivalised total disposable income they receive. It ranges in value from 0, meaning absolute income equality – everyone has the same income – to 1, signalling absolute income inequality – one person has the entire income and all others have none. The income quintile ratio – *S*80/*S*20 ratio – is a measure of the income distribution inequality. It is calculated as the proportion of the total income of 20% of the richest people in society – located in the top quintile – relative to the total income of the 20% poorest people – located in the lowest quintile. Social benefits include all types of monetary social help targeted for poor, disabled, or otherwise handicapped people.

### Methodology

To examine the relationships between these variables, we applied correlation and regression analyses. Through the regression, we quantified the effect of individual income indicators functioning as independent variables on the standardised mortality rate as the dependent variable at a certain significance level.

The general equation used in regression analysis is as follows:

1 $$  Y = \beta_{0} + \sum_{i = 1}^{v} \left ({\beta_{i} X_{i}} \right) + \epsilon  $$

- *Y* – explained variable;
- *β*_0_ – constant;
- *v* – number of explanatory variables;
- *β*_*i*_ – fitted coefficient of the *i*th explanatory variable, whilst $i \in \mathbb {N}$;
- *X*_*i*_ – the *i*th explanatory variable;
- *ε* – residual.

In our analysis, we implemented a modelling process with an outcome in the form of regression models. We applied the linear regression method. To determine the statistical significance of the regression models, four methods were employed – the coefficient of determination and its adjusted version, the bayesian information criterion, and the Akaike information criterion.

The standardised mortality rate *SMR* serves as an explanatory variable. It was constructed for cerebrovascular diseases in particular. The explanatory variables mentioned in the regression analysis are as follows:

- *UR* – unemployment rate;
- *I* – mean net disposable income of a household – expressed in EUR per month;
- *P* – share of population with an income lower than the at-risk-of-poverty threshold in relation to the whole population;
- *GC* – Gini coefficient;
- *IQR* – income quintile ratio;
- *SB* – amount of all social benefits of an individual – expressed in EUR per month.

Mortality rate was expressed as the standardised mortality rate, which is defined as the number of total deaths per 100,000 inhabitants. We applied the method of direct standardisation to eliminate variances resulting from differences in the age structure of the populations across regions and over time, ensuring the necessary conditions for comparing regions of the Slovak Republic.

The standardised cerebrovascular disease mortality rate by sex was calculated for the individual regions of the Slovak Republic during the period from 1996 to 2013 in Microsoft Access using Structured Query Language, and contingency analysis was conducted in Microsoft Excel. Regression analysis was performed in statistical software R. The dataset was in the form of time series panel data combined with cross-sections.

To study the population statistics, we applied descriptive statistical methods, in particular measures of central tendency – minimum, maximum, mean, median and mode – and measures of variability – interquartile range, standard deviation and coefficient of variation.

## Results

### Development of standardised mortality rates for cerebrovascular diseases in the selected European Union members

Cerebrovascular diseases are a leading cause of death in almost all European Union countries, representing approximately 11% of all deaths in these countries [21]. The comparability of the data over time and across different countries was ensured by Eurostat’s Working Group on Public Health Statistics. In contrast to the 2004 to 2010 data, the 2011 to 2012 data were collected with a legal basis [22, 23], however, the comparability of the data was checked before dissemination.

**Fig. 1:**
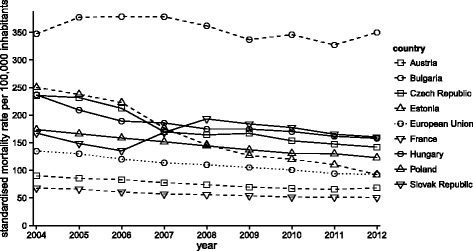
Standardised mortality rate per 100,000 inhabitants for cerebrovascular diseases in the selected countries and in the European Union from 2004 to 2012

Figure 1 depicts the progression in the cerebrovascular disease mortality rate of the European Union countries with the most extreme values throughout the time period from 2004 to 2012. The trend in standardised mortality rate was identified as slightly decreasing, with the exception of the trend in Bulgaria. The highest percentage declines were recorded in countries such as Estonia, where it reached 63.13*%*, and the Czech Republic with a 39.74*%* decline. However, a slight increase at a level of 0.69*%* was revealed in Bulgaria, while the standardised mortality rates were on average 3.2 times higher than the average of the entire European Union. The smallest decline in standardised mortality rate occurred in the Slovak Republic, at a level of 4.23*%*. While the standardised mortality rate of all the observed countries decreased from 2006 to 2008, the Slovak Republic recorded a very sharp increase of 42.7*%*. The average of the entire European Union was characterised by a relatively high decline in the standardised mortality rate for cerebrovascular diseases, reaching 31.5*%*. The Slovak Republic significantly lagged behind the other countries in improvements in the mortality rate within a given time span. In the last year with available statistics, the Visegrad Group members showed standardised mortality rates above the average of the entire European Union. The Slovak Republic had the worst standardised mortality rate at 160 per 100,000 inhabitants, followed by Hungary at 158 per 100,000 inhabitants, the Czech Republic at 142 per 100,000 inhabitants and finally Poland at 132 per 100,000 inhabitants.

### Development of the standardised mortality rate for cerebrovascular diseases in the Slovak Republic

The focus of further analyses was on the standardised mortality rate for cerebrovascular diseases in the Slovak Republic. In the long term, during the period from 1996 to 2013, the trend in standardised mortality rate was cyclical and slightly decreasing.

**Fig. 2:**
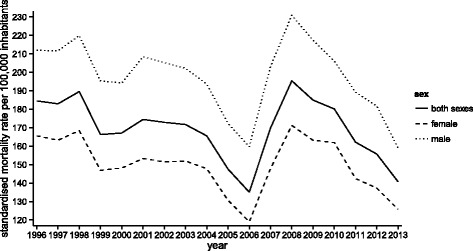
Standardised mortality rate per 100,000 inhabitants for cerebrovascular diseases according to the sexes in the Slovak Republic from 1996 to 2013

The standardised mortality rate for men recorded a 24.99*%* decrease from 211.92 per 100,000 inhabitants in 1996 to 158.96 per 100,000 inhabitants in 2013, compared to a 24.09*%* drop from 165.64 per 100,000 inhabitants in 1996 to 125.73 per 100,000 inhabitants in 2013 for women. Throughout the period from 1996 to 2013, the standardised mortality rate for men was higher by 33% compared to the rate among women. A maximum gender gap was observed in 2007 at a level of 37%, while a minimum difference of 26% was found in 2013, as seen in Fig. 2.

**Fig. 3:**
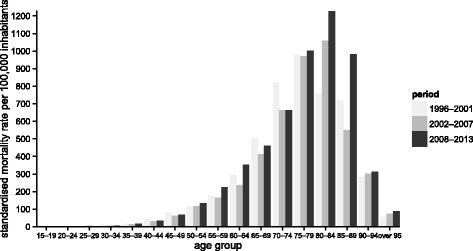
Standardised mortality rate per 100,000 inhabitants for cerebrovascular diseases according to the age groups and the time period in the Slovak Republic from 1996 to 2013

Age plays an important role in the analysis of mortality because it is a significant predictor and an indicator of at-risk age groups. To eliminate fluctuations in the number of deaths, the observed period from 1996 to 2013 was divided into the three periods. Each phase covers 6 years. The first period begins in 1996 and ends in 2001, the second period lasts from 2002 to 2007, and the third period runs from 2008 to 2013 [24]. The number of deaths according to age group, represented by the histogram displayed in Fig. 3, reflects the observations for the three different time periods to detect the age group with the most number of deaths. It shows an exponential growth in the number of deaths up to the group of 75-year-old to 79-year-old people for the first time period and up to the group of 80-year-old to 84-year-old people for the second and also the third period. Regarding old age mortality, negative linear trends in frequencies are demonstrated.

The median standardised mortality rate was set at the 75-year-old to 79-year-old age group, representing 50% of deaths above this age group. In relation to the advanced age of death, it can be supposed that the background or origin of cerebrovascular diseases is chronic in many cases of death. The interquartile range lies between the 65-year-old to 69-year-old and 80-year-old to 84-year-old age groups, representing the age characteristics of half of the deaths in the period from 1996 to 2001. From 2002 to 2013, the first quartile transitioned to the 70-year-old to 74-year-old age group and the third quartile did not change, and thus a 50% share of deaths was narrowed down to the age group of 70-year-old to 84-year-old people.

The results reveal a decreased level of premature mortality for cerebrovascular diseases, which was typically represented by deaths up to the 75th year of age [25]. This finding may be partly related to the ageing of the Slovak population as well as to an increase in life expectancy at birth from 77.5 years in 2000 to 79.9 years in 2012. Therefore, in the following analysis, the values of the standardised mortality rate are considered to eliminate bias. For a deeper analysis of the mortality caused by cerebrovascular diseases in the Slovak Republic, it was desirable to examine regional differences based on sex and using a long-term approach.

### Regional differences in the development of standardised mortality rate for cerebrovascular diseases in the Slovak Republic

Based on the Nomenclature of Units for Territorial Statistics geocode standard, the Slovak Republic is divided into 8 geographic regions: the Banská Bystrica Region, the Bratislava Region, the Košice Region, the Nitra Region, the Prešov Region, the Trencín Region, the Trnava Region, and the žilina Region.

As seen in Table 1, in terms of the individual regions of the Slovak Republic for men, the standardised mortality rates range from 143.42 per 100,000 inhabitants in the Prešov Region to 288.73 per 100,000 inhabitants in the Banská Bystrica Region between 1996 and 2001; they further vary from 108.57 per 100,000 inhabitants in the Bratislava Region to 237.10 per 100,000 inhabitants in the Nitra Region in the period from 2002 to 2007, and finally from 113.05 per 100,000 inhabitants in the žilina Region to 241.8 per 100,000 inhabitants in the Nitra Region during the last period from 2008 to 2013.

**Table 1:** Standardised mortality rate per 100,000 inhabitants for cerebrovascular diseases for men in the regions of the Slovak Republic according to the time periods

Region	1996–2001		2002–2007			2008–2013			Coefficient of variation
	SMR	Rank	SMR	Rank	Change	SMR	Rank	Change	
BC	288.73	8th	218.83	7th	−24.21*%*	208.01	5th	−4.94*%*	20.58
BL	170.40	2nd	108.57	1st	−36.29*%*	113.05	1st	4.13*%*	26.21
KI	187.03	4th	207.93	5th	11.18*%*	209.64	6th	0.82*%*	22.71
NI	236.62	7th	237.10	8th	0.20*%*	241.80	8th	1.98*%*	15.90
PV	143.42	1st	151.15	2nd	5.39*%*	202.30	4th	33.84*%*	24.99
TA	231.51	6th	214.40	6th	−7.39*%*	217.46	7th	1.43*%*	15.30
TC	208.38	5th	191.49	4th	−8.10*%*	192.04	2nd	0.29*%*	16.50
ZI	183.84	3rd	181.14	3rd	−1.47*%*	196.01	3rd	8.21*%*	14.90

Legend: *SMR* standardised mortality rate, *BC* the Banská Bystrica Region, *BL* the Bratislava Region, *KI* the Košice Region, *NI* the Nitra Region, *PV* the Prešov Region, *TA* the Trnava Region, *TC* the Trencín Region, *ZI* the žilina Region

Note: Change is computed as period-over-period change

Source: based on own elaboration by the authors

Table 2 reflects the descriptive statistics of standardised mortality rate in each time period. Throughout the entire explored time span, the median standardised mortality rate for men increased from 197.70 per 100,000 inhabitants to 205.15 per 100,000 inhabitants, representing a deterioration in mortality accompanied by considerable period-over-period increases in the Prešov Region, at levels of 5.39*%* and 33.84*%*; in the Košice Region, at levels of 11.18*%* and 0.82*%*; and in the Nitra Region, at levels of 0.20*%* and 1.98*%* between each two successive periods, respectively, as seen in Table 1. In all three time periods, the Banská Bystrica Region, the Nitra Region, and the Trnava Region had a standardised mortality rate above the median for men, while the Bratislava Region and the žilina Region consistently showed values lower than the median. Although the standardised mortality rate attained in the Banská Bystrica Region was high, this region also showed the greatest progress in improving mortality results. The reduced variability in standardised mortality rate for cerebrovascular diseases was confirmed by the values of standard deviation as well as the coefficient of variation, which recorded a downward trend throughout the entire time span, as displayed in Table 2. As for the variability of each region, the highest value was achieved in the Bratislava Region, at a level of 26.21. The lowest value, 14.90, was observed in the žilina Region. Although the Bratislava Region could be considered the best in terms of mortality, the variability results showed quite high volatility in positive results in this region.

**Table 2:** Descriptive statistics of standardised mortality rates for cerebrovascular diseases for men in the Slovak Republic

Indicator	1996–2001	2002–2007	2008–2013
Minimum	143.42	108.57	113.05
Maximum	288.73	237.10	241.80
Median	197.70	199.71	205.15
Mean	206.24	188.82	197.54
Standard deviation	45.47	41.71	37.43
Coefficient of variation	22.05	22.09	18.95

Source: based on own elaboration by the authors

The standardised mortality rate was an average of 33% lower for women than for men. During the period from 1996 to 2001, its values for women range from 110.44 per 100,000 inhabitants in the Prešov Region to 227.15 per 100,000 inhabitants in the Banská Bystrica Region. From 2002 to 2007, the minimum value increased only to 83.79 per 100,000 inhabitants in the Bratislava Region. Conversely, the maximum value peaks at a level of 183.33 per 100,000 inhabitants in the Nitra Region. In the period from 2008 to 2013, the Bratislava Region with a value of 89.78 per 100,000 inhabitants remained the best, compared with the Nitra Region at a level of 183.33 per 100,000 inhabitants, representing the worst mortality rate of all regions. According to Table 3, the percentage changes in the standardised mortality rate for women expose relatively large differences between the regions. In the period from 2002 to 2007, a majority of the regions recorded a decline in percentage compared with the period from 1996 to 2001. The highest percentage decreases were recorded in the Bratislava Region, at a value of 34.64*%*; in the Banská Bystrica Region, with a value of 29.94*%*; and in the Trencín Region, with a value of 17.44*%*.

**Table 3:** Standardised mortality rate per 100,000 inhabitants for cerebrovascular diseases for women in the regions of the Slovak Republic according to the time periods

Region	1996–2001		2002–2007			2008–2013			Coefficient of variation
	SMR	Rank	SMR	Rank	Change	SMR	Rank	Change	
BC	227.15	8th	159.14	6th	−29.94*%*	167.45	6th	5.22*%*	21.38
BL	128.20	2nd	83.79	1st	−34.64*%*	89.78	1st	7,16*%*	25.69
KI	141.56	4th	149.35	5th	5.51*%*	150.54	4th	0.79*%*	22.05
NI	171.61	7th	183.33	8th	6.83*%*	183.33	8th	0%	17.91
PV	110.44	1st	113.88	2nd	3.11*%*	152.71	5th	34.10*%*	25.87
TA	170.73	6th	161.75	7th	−5.26*%*	170.67	7nd	5.51*%*	14.12
TC	165.27	5th	136.44	4th	−17.44*%*	146.02	3th	7.02*%*	16.69
ZI	135.01	3rd	133.69	3rd	−0.98*%*	142.38	2rd	6.50*%*	15.33

Legend: *SMR* standardised mortality rate, *BC* the Banská Bystrica Region, *BL* the Bratislava Region, *KI* the Košice Region, *NI* the Nitra Region, *PV* the Prešov Region, *TA* the Trnava Region, *TC* the Trencín Region, *ZI* the žilina Region

Note: Change is computed as period-over-period change

Source: based on own elaboration by the authors

Similarly as seen in Table 4, the median standardised mortality rate falls from 153.41 per 100,000 inhabitants to 142.90 per 100,000 inhabitants. In the last time period, the median increases to 151.63 per 100,000 inhabitants. This trend was accompanied by an increase in the growth rate of standardised mortality rate in all regions except for the Košice and Nitra Regions. However, the most growth occurred in the Prešov Region, at a level of 34.10*%*. The Nitra Region showed no change in growth rate, although it increased during the entire examined time span in the Košice Region as well as in the Prešov Region. The Banská Bystrica Region, Nitra Region, and Trnava Region had a standardised mortality rate above the median, while the Bratislava Region and the žilina Region consistently showed values lower than the median throughout the whole time span. The standard deviation as well as the coefficient of variation showed a downward trend. As for women, the rates of variability gained the highest values in the Prešov Region, at a level of 25.87, and in the Bratislava Region, at a level of 25.69. On the contrary, the lowest values are found in the Trnava Region, at a value of 14.12, and in the žilina Region, reaching a value of 15.33.

**Table 4:** Descriptive statistics of standardised mortality rates for cerebrovascular diseases for women in the Slovak Republic

Indicator	1996–2001	2002–2007	2008–2013
Minimum	110.44	83.79	89.78
Maximum	227.15	183.33	183.33
Median	153.41	142.90	151.63
Mean	156.25	140.17	150.36
Standard deviation	36.07	30.93	28.16
Coefficient of variation	23.08	22.07	18.73

Source: based on own elaboration by the authors

**Fig. 4:**
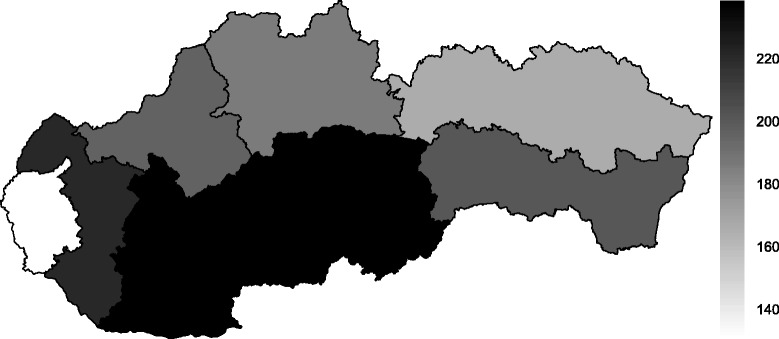
Average level of standardised mortality rate per 100,000 inhabitants for cerebrovascular diseases for men in the regions of the Slovak Republic from 1996 to 2013

**Fig. 5:**
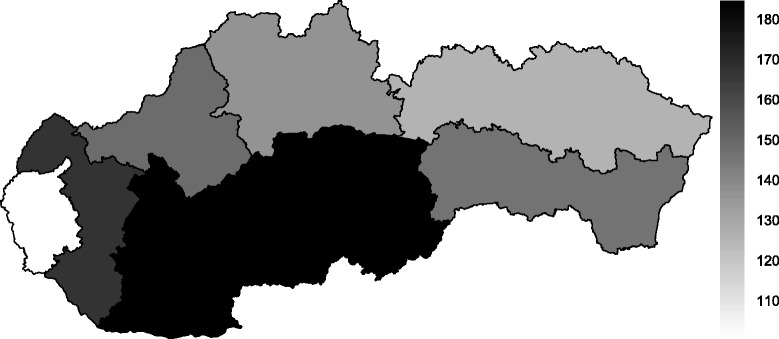
Average level of standardised mortality rate per 100,000 inhabitants for cerebrovascular diseases for women in the regions of the Slovak Republic from 1996 to 2013

To clearly show the regional disparities, Figs. 4 and 5 represent the status of the Slovak regions in terms of their average standardised mortality rates for cerebrovascular diseases during the whole time span, both for men and women. The worst values were observed in the southern regions – namely, the Nitra Region and the Banská Bystrica Region. In contrast, better results were associated with northern Slovakia – the žilina Region and the Prešov Region – and the most favourable standardised mortality rate for cerebrovascular diseases occurred in the Bratislava Region. It is remarkable to observe the northern regions with lower standardised mortality rates and the southern regions with higher standardised mortality rates. These differences likely relate to the risk factors, the socio-economic indicators and the environmental factors influencing cerebrovascular diseases in these individual regions.

### Regression analysis

The examined models are presented in Table 5. Fitted coefficients and *p*-values are displayed for each variable involved in the particular model.

**Table 5:** Beta coefficients

Coefficient	*M*_1_	*M*_2_	*M*_3_	*M*_4_
*β*_0_			−410.606	−594.3626
*UR*	0.0659	0.0587	0.2213	0.0551
*I*	0.0819	0.0134	0.8793	
*P*	0.2529		0.68	0.0279
*GC*	0.0206	0.0042	0.3616	0.0315
*IQR*	0.0113	0.0034	0.4439	0.0269
*SB*	0.0784	0.0659	0.2365	0.0654

Source: based on own elaboration by the authors

The following Table 6 demostrates the quantified standardised beta coefficients for the explanatory variables of the regression models.

**Table 6:** Standardised beta coeffcients

Variable	*M*_1_	*M*_2_	*M*_3_	*M*_4_
*β*_0_			2.2240×10^−15^	2.6543×10^−15^
*UR*	−2.1068	−1.9361	−2.1068	−2.1489
*I*	−1.0149	−3.8777	−1.0149	
*P*	1.7317		1.7317	2.3268
*GC*	9.8444	6.5488	9.8444	10.927
*IQR*	−10.1619	−5.5587	−10.162	−11.709
*SB*	1.3940	1.3006	1.3940	1.4170

Source: based on own elaboration by the authors

The subsequent Table 7 visualises significance of the quantified variables involved in the regression models in form of *p*-value.

**Table 7:** Significance of variables

Variable	*M*_1_	*M*_2_	*M*_3_	*M*_4_
*β*_0_			0.7488	0.072
*UR*	0.0659	0.0587	0.2213	0.0551
*I*	0.0819	0.0134	0.8793	
*P*	0.2529		0.68	0.0279
*GC*	0.0206	0.0042	0.3616	0.0315
*IQR*	0.0113	0.0034	0.4439	0.0269
*SB*	0.0784	0.0659	0.2365	0.0654

Source: based on own elaboration by the authors

The first model series represents a model set expressing the standardised mortality rate by all the variables except for the constant value. The first two models – *M*_1_ and *M*_2_ – were part of this series. In the first step, the poverty indicator was deleted from the modelling process as the variable with the worst *p*-value. The best model in this series was represented by the second model, *M*_2_.

The *M*_2_ model explained the standardised mortality rate as follows:

2 $${} \begin{aligned} M_{2} = &-1.9361 UR - 3.8777 I + 6.5488 GC - 5.5587 IQR\\ &+ 1.3006 SB \end{aligned}  $$

The coefficient of determination of this model reached a value of 0.9557, and although the adjusted coefficient of determination declined to 0.3584, the dataset can be considered well fitted by the model *M*_2_. The model’s *p*-value for F statistics was 0.0311. The choice of the second model *M*_2_ was also confirmed by the bayesian information criterion, which reached −5.10 in the model *M*_2_, whereas the corresponding value for the first model *M*_1_ was −2.97. The Akaike information criterion further confirmed this situation, as its value for the first model *M*_1_ was 297.13 and was 240.41 for the second model *M*_2_. Moreover, all the included variables fulfilled at least the ten-percent significance level with only two dimensions – unemployment rate and social benefits – slightly overstepping the five-percent significance level. The Gini coefficient had the largest impact on mortality rate, with a beta coefficient reaching 6.5488. The income quintile share ratio had the next largest effect, with a value of −5.5587, followed by income with a value of 3.8777, unemployment rate with a value of −1.9361 and finally social benefits with a value of 1.3006.

The second model series was based on the previous one with only one alternation – a constant value in the form of the intercept was added. In the first step, income was removed from the modelling process because its *p*-value was the highest of all the involved variables. The second model of the series was again the best, although it cannot be taken into consideration, because only the correct constant value would remain in the successive step of the modelling process.

3 $${} \begin{aligned} M_{4} &= 2.6543 \times 10^{-15} -\! 2.1489 UR +2.3268P +\! 10.927 GC \\ &\quad - 11.709 IQR + 1.4170 SB \end{aligned}  $$

Model *M*_4_ fits the dataset well; this was confirmed by the coefficient of determination, which reached a value of 0.9824, and its adjusted version, showing a value of 0.2456. The model itself fulfilled the five-percent significance level, with a *p*-value for F statistics of 0.0433. Therefore, continuing this modelling process was pointless. The bayesian information criterion expressed the same result – the value for the third model *M*_3_ was −0.55, whereas it was −2.97 for the fourth model *M*_4_. The Akaike information criterion further confirmed this situation – the third model *M*_3_ had a value of 231.50 and the fourth model *M*_4_ a value of 231.00.

## Discussion

The variability of the standardised mortality rate gradually declined during the given time periods. The worst standardised mortality rates were recorded in the Banská Bystrica Region as well as in the Nitra Region, and the best value was recorded in the Bratislava Region throughout the explored time span. The standardised mortality rate values were lower in the northern part of the Slovak Republic compared with the southern part of the country. However, the Bratislava Region and also the Prešov Region showed the highest variability in standardised mortality rate. In contrast, the lowest variability was typical for the žilina Region and the Trnava Region. Although the Bratislava Region was considered the best in terms of mortality rate, the variability results in this region demonstrated high volatility in positive results. However, the žilina Region showed a high level of stability of positive results regarding mortality rate. As for men, only the Banská Bystrica Region showed a permanent percentage drop in standardised mortality rate between the observed time periods, while the opposite tendency was indicated in the Košice Region, the Nitra Region, and in the Prešov Region. The other regions demonstrated a volatile development rate. As for women, a permanent percent increase in standardised mortality rate was observed in the Košice Region and Prešov Region.

The regression analysis revealed several dimensions that had an impact on standardised mortality rate. Of all the examined variables, unemployment rate, household disposable income and income quintile ratio had a negative impact, helping to reduce the mortality rate. However, at-risk-of-poverty status, Gini coefficient and social benefits positively influenced mortality rate. All these dimensions appeared statistically significant and reliably described the standardised mortality rate.

From the perspective of the indicators examined in this study, mortality rate can be described by unemployment rate, household disposable income, share of at-risk-of-poverty population, Gini coefficient, income quintile ratio, and social benefits. This finding was statistically confirmed. The factor with the largest influence was income quintile ratio, which had the highest beta coefficient in the model M_4_ that can be regarded as being the most meaningful model than others, since the second model series - the models M_3_ and M_4_ - contains the intercept *β*_0_. Based on this finding, we suggest considering income quintile ratio within the Slovak population when arranging out-of-pocket payments to ensure that low-income groups are not at risk due to high payments for delivered health care.

Slovak men recorded higher values of standardised mortality rate by nearly 33% compared with women. However, an average decline of 24% in standardised mortality rate was observed for both sexes from 1996 to 2013. The reason for the sharp increase in mortality from 2006 to 2008 is described in a study by Hlavatý and Liptáková [26]. They revealed that in 2005 and 2006, the absolute number of deaths for cerebrovascular diseases, marked as *I*60 to *I*69 according to the World Health Organization’s International Statistical Classification of Diseases, was undervalued by 50% in favour of deaths for hypertension, which were marked as *I*10 to *I*15, due to an incorrect coding of the causes of death in statistical processing. After 2006, the National Health Information Centre conducted a revision of the coding, leading to a sharp increase in deaths from cerebrovascular diseases. Since 2007, all causes of deaths have been coded according to international recommendations by Eurostat and the World Health Organization documented in the Manual on the certification of causes of death in Europe [27]. The greatest difference between men and women occurred in 2007, while the smallest gap was revealed in 2013. The incidence of mortality for cerebrovascular diseases has shifted to higher ages over the years. People at the highest risk were in the age group of 70 to 84 years from 1996 to 2001 and from 75 to 84 years from 2002 to 2013.

A limitation of this study is the lack of availability of data on individual income level in the mortality database, and thus summary measures for income indicators in each region were applied.

The variability in mortality rate development in each year shows that the development of mortality should also be examined in terms of regional disparities to reflect the factors strongly contributing to the development in mortality rate in individual regions [28, 29]. By mapping regional disparities, prevention programmes and other interventions that could regulate mortality in individual regions can be effectively established. By implementing active prevention programmes targeted to selected population groups as well as to particular regions, mortality and morbidity can be actively controlled, and these programs also contribute to increase the effectiveness of the health system [30, 31].

Many educational activities devoted to prevention programmes for cerebrovascular disease risk factors are clearly priorities of the health policy in the Slovak Republic, namely the Monika project [32], the Cindi project [33], the National Programme of Prevention Heart Conditions in Adults [34], and the National Action Plan for the prevention of obesity for the years 2015–2025 [35]. Their aim is to ensure effective long-term education of the population at all societal levels.

As for international comparisons, the standardised mortality rate for cerebrovascular diseases in the Slovak population showed alarming values in comparison with the entire European Union average. Many studies [36–38] have shown that there is a diversity of health policy approaches to reducing the incidence of risk factors affecting cerebrovascular diseases, such as unhealthy lifestyles, smoking, and obesity as well as lower access to health care associated with the population’s socio-economic status.

Carefully prepared mortality analyses can provide a valuable platform for developing the methodology of avoidable mortality, which currently is solely dependent on the health systems of interest to its creators [24, 39, 40]. The results of the available methodologies of avoidable mortality [41] warrant caution in their interpretation because each one has a specific methodology and inclusion or exclusion diagnoses. According to many authors [8, 42, 43], mortality provides a reliable picture of public health and is also the most objective way of measuring health.

## Conclusion

To conclude, at the present time, the mortality rate for cerebrovascular diseases has decreased in many European Union countries as well as in Slovakia.

Mortality is characterised by a relatively large amount of inertia in its development, and therefore, it is not expected that the described differences between the Slovak Republic population and that of the other European countries will be diminished in the next few years. In our study, we present an evaluation of the development of mortality rates for cerebrovascular diseases in the Slovak Republic. In addition, our objective was to quantify the regional disparities and to analyse the development of the mortality rate in relation to income inequalities in the individual regions of the Slovak Republic. Income quintile ratio appears to be the most influencing dimension in these models of standardised mortality rate. Considering the process of demographic ageing as well as the increase in the number of older people in the European Union and worldwide, responsibility for health should be prioritised.
